# Engineering Nitrogenases for Synthetic Nitrogen Fixation: From Pathway Engineering to Directed Evolution

**DOI:** 10.34133/bdr.0005

**Published:** 2023-02-07

**Authors:** Emily M. Bennett, James W. Murray, Mark Isalan

**Affiliations:** Department of Life Sciences, Imperial College London, London SW7 2AZ, UK.

## Abstract

Globally, agriculture depends on industrial nitrogen fertilizer to improve crop growth. Fertilizer production consumes fossil fuels and contributes to environmental nitrogen pollution. A potential solution would be to harness nitrogenases—enzymes capable of converting atmospheric nitrogen N_2_ to NH_3_ in ambient conditions. It is therefore a major goal of synthetic biology to engineer functional nitrogenases into crop plants, or bacteria that form symbiotic relationships with crops, to support growth and reduce dependence on industrially produced fertilizer. This review paper highlights recent work toward understanding the functional requirements for nitrogenase expression and manipulating nitrogenase gene expression in heterologous hosts to improve activity and oxygen tolerance and potentially to engineer synthetic symbiotic relationships with plants.

## Introduction

Major crops such as wheat and maize depend on bioavailable nitrogen in the soil for growth, and the use of nitrogen-containing fertilizer results in dramatic increases in crop yield. Indeed, without chemical fertilizers, produced by industrial nitrogen fixation, the world could only support 3.5 billion people [1], rather than the current 8 billion.

This review will examine the genetic components that synthetic biologists are using to fix nitrogen, while considering how natural systems have evolved to achieve this. It will also explore how these components are being engineered into bacterial hosts, looking at problems such as tuning component stoichiometries for nitrogenase complexes, controlling gene regulation, and engineering oxygen protection and symbiotic plant–microbe relationships. Finally, it will consider how powerful methods such as directed evolution might be applied to optimize such processes in a heterologous context.

### Genetic components and mechanism of nitrogen fixation

**Fig. 1.**
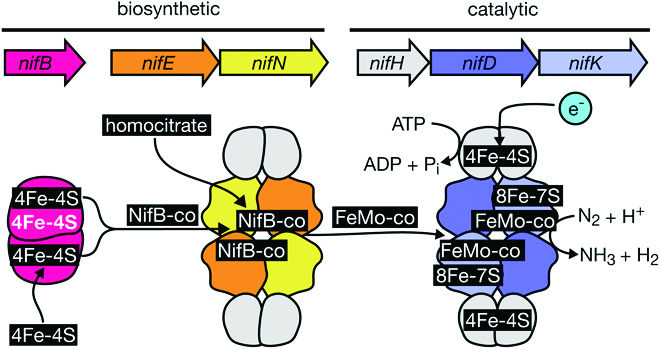
Minimum set of *nif* genes essential for nitrogen fixation with molybdenum-iron nitrogenase. Please note that shown stoichiometry has not been adjusted. NifB contains one catalytic cluster (shown in white) and 2 substrate [4Fe-4S] clusters that react to produce the NifB cofactor. NifEN matures the NifB cofactor producing the FeMo cofactor. The molybdenum-iron (MoFe) nitrogenase (NifHDK) contains the FeMo cofactor at its active site. Electron donors transfer single electrons to the [4Fe-4S] cluster at the interface of the NifH homodimer. Electrons are moved from the [4Fe-4S] cluster into the active site of nitrogenase using energy produced by ATP hydrolysis by NifH. A minimum of 8 electrons are used to reduce each molecule of N_2_.

In the biological nitrogen fixation reaction, N_2_ is reduced to form NH_3_ under ambient conditions by the nitrogenase enzyme [2]. Nitrogen fixation (N_2_ + 8H^+^ + 8e^-^ + 16MgATP ➔ 2NH_3_ + H_2_ + 16MgADP + 16P_i_) requires a source of ATP, protons, and electrons. *Nif* gene clusters, encoding nitrogenase and numerous associated enzymes, occur in a diverse set of prokaryotes including bacteria and archaea [3]. Nitrogen fixation itself occurs through the coordinated activities of homodimeric NifH and the heterotetrametric NifDK complex (Fig. 1). Reduced electron carriers donate electrons to the [4Fe-4S] cluster at the interface of the NifH homodimer. Electron carriers are typically flavodoxins and ferredoxins, depending on the organism. During the catalytic cycle, NifH transiently binds and then dissociates from the NifDK complex, hydrolyzing 2 molecules of Mg-ATP per electron transfer. Electrons are sequentially donated to the [8Fe-7S] cluster in the NifDK protein, then finally to FeMo-co (a [Mo-7Fe-9S-C-homocitrate cluster], co: cofactor), which binds and reduces N_2_ to form NH_3_ and H_2_ [4].

The primary enzyme responsible for nitrogen fixation is the molybdenum (Mo)-dependent nitrogenase, encoded by the genes *nifH*, *nifD*, and *nifK*. Some prokaryotes also contain *Anf* or *Vnf* genes encoding alternative nitrogenases, which have only iron (Fe) or vanadium (V) and iron in their active sites. These alternative nitrogenases have different gene requirements; MoFe nitrogenase requires *nifEN* and VFe nitrogenase requires *vnfEN*. However, there is no *anfEN*, and the FeFe cluster is inserted directly onto the AnfDGK scaffold [5]. FeFe (AnfHDGK) and VFe (VnfHDGK) nitrogenases have lower nitrogenase activity than MoFe (NifHDK) nitrogenases, and therefore, MoFe nitrogenase is likely preferred in nature, except in molybdenum-scarce environments [6]. All genomes containing FeFe and VFe nitrogenases also encode MoFe nitrogenase. They are also more O_2_-sensitive than MoFe nitrogenases and produce more H_2_ per reaction [7]. Furthermore, some assembly factors for assembly of the FeMo cofactor (typically NifU, NifS, NifV, NifB, and NifM) are also required for the assembly of the alternative nitrogenases [8]. Therefore, for synthetic engineering projects, MoFe nitrogenases are the preferred scaffold for engineering nitrogen fixation in heterologous hosts.

Nitrogen-fixation chemistry depends on enzymes containing iron-sulfur (Fe-S) clusters. Fe-S clusters can accept and donate electrons, making them essential components of cellular processes spanning from gene regulation, to electron transfer in biosynthesis, to metabolism [9,10]. NifS and NifU are the most common nitrogenase-specific Fe-S cluster biosynthesis enzymes; however, other enzymes from the *isc* and *suf* gene families also participate in Fe-S biosynthesis [9]. Enzymes responsible for the synthesis and assembly of Fe-S clusters and FeMo-co are often co-located with nitrogenase structural genes in gene clusters. Of these enzymes, NifB and NifEN are thought to be the only *nif*-specific factors that are absolutely required for assembly of active nitrogenase, and whose activities cannot be substituted by other host cell enzyme activities. NifB is an *S*-adenosylmethionine-dependent enzyme responsible for the rearrangement and catalytic conversion of two [4Fe-4S] clusters to a [8Fe-9S-C] cluster called NifB-co; this, in turn, is bound and transferred to NifEN by NifX [11]. NifV synthesizes the homocitrate in the active site cofactor [12]. Fe-S clusters are also highly sensitive to damage by reactive oxygen species, which results in the oxygen sensitivity of many enzymes with Fe-S cofactors [13]. The major mechanism of oxygen damage is reactivity of the surface-exposed [4Fe-4S] cluster in NifH and the FeMo cofactor in NifDK heterotetramer.

The fact that nitrogenase is irreversibly damaged by molecular oxygen is a key hurdle for nitrogenase synthetic biology. This oxygen sensitivity limits both the throughput of nitrogenase-focused engineering projects (anaerobic conditions complicate experimental setup) and the activity of heterologous nitrogenase genes under aerobic or microaerobic conditions. Diazotrophic organisms therefore employ several mechanisms of oxygen protection: spatial and temporal separation of *nif* gene expression, physical protection, conformational protection, and respiratory protection [13]. Nitrogenase activity that can withstand aerobic or microaerobic environments is a highly desirable feature for bioengineering; therefore, synthetic biologists may employ any one of these mechanisms to protect heterologous nitrogenase genes from oxygen. Notably, both oxygen and fixed nitrogen are strong regulators of *nif* gene expression in nitrogen-fixing species. Manipulating *nif* gene regulation allows us to balance expression from engineered nitrogenase gene clusters, which is an important prerequisite to survival of engineered species in the soil environment.

### Genetic regulation of *nif* clusters

Engineered *Nif* clusters may use either constitutive or responsive expression, in which nitrogenase gene expression depends on intracellular conditions or external chemical signals. Natural *nif* gene expression is tightly regulated by transcription factors in response to molecular oxygen, cellular energy levels (including carbon and ATP availability), and nitrogen availability [13]. In proteobacteria, including representative species *Klebsiella oxytoca*, *nif* gene expression is dependent on the alternative sigma factor, σ^54^. Transcriptional initiation from σ^54^ promoters affects the expression of hundreds of genes, with diverse functions, including ammonia import, through to signaling and metabolism [14–16]. Below are some examples of *nif* gene regulation.

In *K. oxytoca*, expression of *nif* genes depends on intracellular nitrogen availability and is linked to the nitrogen-starvation response pathway, through the NtrB/NtrC two-component transcriptional activator. The latter binds and activates transcription from σ^54^ promoters under conditions of nitrogen stress (low cellular fixed nitrogen) [14]. The activated promoters include the *nifLA* operon, which links *nif* gene expression to cellular nitrogen status. Binding of GlnK to NifL promotes dissociation of NifL from NifA, which binds to and activates expression of catalytic *nif* genes [13,17].

In *Azotobacter vinelandii*, regulation in response to fixed nitrogen occurs at the protein level; NifA is activated posttranslationally by GlnK and 2-oxoglutarate binding, resulting in dissociation of NifA from NifL and subsequent activation of downstream promoters for *nif* genes. In both these representative species, GlnK integrates signals from the carbon and nitrogen status of the cell, ensuring that expression only occurs when (a) the cell requires fixed nitrogen and (b) ATP production from metabolism is able to support the ATP requirement of nitrogenase [18].

While *Paenibacillus* species do not include a *nifA* homolog, GlnR is proposed to be both an activator and a repressor, activating transcription of *nif* genes under conditions of nitrogen starvation, and repressing transcription under conditions of nitrogen surplus [19]. In *Paenibacillus polymyxa*, a free-living soil nitrogen-fixing soil bacterium, *nifH* and *nifK* are expressed at high levels under low fixed nitrogen and oxygen concentrations (<2 mM NH_4_^+^ and <5% O_2_) concentrations. These expression profiles match the activity profile of nitrogenase under different oxygen concentrations, thereby ensuring that nitrogenase is not expressed under conditions where it would not be active [20]. Such conditional expression of *nif* genes could be mimicked in a laboratory project, to create diazotrophy in a model organism such as *Escherichia coli*, preventing metabolically expensive nitrogenase expression under higher-oxygen conditions where it would be inactive.

The high metabolic cost of *nif* gene expression and the necessary cofactor and [Fe-S] cluster biosynthesis means that each *nif* gene included in an engineered *nif* construct must be carefully considered to identify the smallest or minimal gene set that will fix nitrogen. Smaller, less complex clusters are advantageous for engineering because they have a smaller combinatorial “expression space” of levels of nitrogenase proteins to explore, making them easier to rationally optimize.

### Minimal *nif* clusters

One important hurdle to engineering nitrogenase is the complexity of *nif* clusters, leading many researchers to propose a minimal set of genes that are essential for nitrogen fixation (Fig. 1), while supplementing other required activities with native host genes [5,20]. Proposed minimal sets of genes include genes encoding nitrogenase core catalytic enzymes (*nifHDK*), in addition to host-specific catalytic and biosynthetic genes, which are essential for activity. The absolute requirement for genes encoding synthesis enzymes is host-dependent, as some enzymatic synthesis activities, for example, synthesis of Fe-S or homocitrate, can be partially or completely substituted by native host genes in some nondiazotrophic organisms [21]. Furthermore, nitrogenase is compatible with alternative ferredoxins and flavodoxins only in some hosts, with varying electron transfer efficiencies [22]. Incompatible or partially incompatible electron carriers may limit or completely obliterate nitrogenase activity; therefore, additional electron carrier genes may be required for activity in some organisms. One definition of what comprises the minimal *nif* cluster includes only genes that encode catalytic (e.g., *nifHDK*) and enzymatic activities (e.g., *nifENB*) that are unique to nitrogenase, such as *nifHDKENB* [8]. The Fe-only (AnfHDGK) nitrogenase does not require a NifEN homolog for cofactor maturation, offering the opportunity to further simplify nitrogenase cassettes by excluding *nifEN* [5]. Ancestral sequence reconstruction of catalytic nitrogenase gene ancestors, or the putative NifD/NifK homodimeric ancestor, could also be used to simplify synthetic gene clusters or engineer improved nitrogenase variants. Genes that have been reconstructed from inferred evolutionary ancestors are considered more “evolvable” than their modern homologs and therefore may be a useful starting point for directed evolution [23]. Understanding the minimal requirements for functioning of nitrogenase in heterologous systems is crucial, allowing us to build nitrogenase activity from the bottom-up with the tools of synthetic biology. The next section will focus on the current state of the art in nitrogenase engineering and discuss avenues for further investigation.

## Engineering Nitrogenase Clusters in Native and Heterologous Hosts

Efforts to engineer synthetic nitrogenase activity focus on a couple of key challenges: the expression of correctly folded, highly active, oxygen-protected *nif* gene products in native and novel hosts such as bacteria or plants and improving the compatibility of heterologous nitrogenase clusters with their hosts.

Directed evolution may be used to optimize expression levels by mutating regulatory elements or the gene coding sequences themselves to enhance activity, solubility, or stability in different host contexts.

### Starting points: Portability of *nif* clusters between species

Nitrogen fixation activity was first transferred to *E. coli* in 1972 from *K. oxytoca*, a gram-negative bacterium that forms symbiotic associations with many plants [24]. Since the 1970s, many *nif* genes from diazotrophic species have been successfully transferred into *E. coli* [20,25–27]. *E. coli* does not itself contain *nif* genes or fix nitrogen and is a well-studied model organism amenable to genetic manipulation. Tuning and measuring activity of nitrogenase genes in *E. coli* can reveal the important factors for nitrogenase engineering, which can be applied to reconstruct optimal *nif* gene cassettes for a desired host.

*Klebsiella* species contain large multi-operon *nif* clusters, which often have high nitrogenase activity when transferred to *E. coli*. Therefore, these are a useful starting point for improving nitrogen fixation in *E. coli. Azotobacter* have also been of particular interest, as they are able to fix nitrogen in aerobic environments, through many known (and unknown) mechanisms [28]. The smallest *nif* operon discovered to date, the *P. polymyxa* sp. *WLY78*, comprises just 9 genes in a single operon, which enables low but detectable (10%) nitrogen fixation activity when transferred into *E. coli* [20]. Activity was reduced by 50% when either *nifX* (nitrogenase assembly factor) or *hesA* (unknown function) was deleted, indicating that these gene functions are required in *E. coli* for nitrogen fixation activity [20]. Smaller or minimal gene clusters such as this one are simpler to understand and engineer, as they have reduced expression space dimensionality compared to larger clusters. However, they may be more fragile than larger clusters and therefore have lower activity than larger clusters (such as those from *Klebsiella* or *Azotobacter*), even when fully optimized.

There has also been considerable interest in engineering bacteria that associate with cereals to fix nitrogen. This approach is attractive because symbiosis is considered at least as difficult to engineer as nitrogen fixation, as it may require manipulation of both the plant and the engineered bacteria to establish cooperation or nutrient dependency, and it is mechanistically less well understood [29]. Transfer of *nif* genes from *Pseudomonas stutzeri* to *Pseudomonas protegens* Pf-5, a soil bacterium that could be used to colonize cereal crop roots, resulted in a remarkably high nitrogenase activity and permitted diazotrophic growth using only N_2_ as a nitrogen source [30]. Nitrogenase activity in the engineered strain was found to be insensitive to exogenous ammonia concentration and oxygen, indicating that the regulation mechanisms were sufficiently different between the two species to escape regulation [30]. The engineered strain also excreted additional ammonia into the soil and increased growth of *Arabidopsis* and *Medicago sativa* in soil containing low amounts of fixed nitrogen. While such results do not show survival in a realistic agricultural environment, they propose that introduction of a deregulated strain into the environment may temporarily increase crop growth. Unfortunately, it is likely that such a bacterium in an agricultural context would be at a competitive disadvantage compared to other soil bacteria, eventually leading to elimination through competition. Nevertheless, this study was fundamental in promoting alternative chassis strains for synthetic nitrogen fixation.

Building on this work was a landmark study where 12 *nif* clusters from diverse species were transferred into *E. coli MG1655*, *P. protogens Pf-5*, and rhizobia bacteria, with the aim of identifying bacteria amenable to genetic engineering that can form associations with cereal crops. *E. coli* showed the most flexibility in accepting foreign nitrogenase genes, with 7 out of the 10 tested clusters showing activity. Results for transfers into other species were more mixed; many showed no detectable nitrogenase activity. Many of these clusters retained native regulatory patterns, most notably repression by excess nitrogen in the media, indicating that their promoters were connected to the native nitrogen starvation response by transcriptional activation through the action of the exogenous *nifLA* genes [31]. Additional modifications of these clusters and their effects will be discussed in the relevant sections below.

### Nitrogenase overexpression

**Fig. 2.**
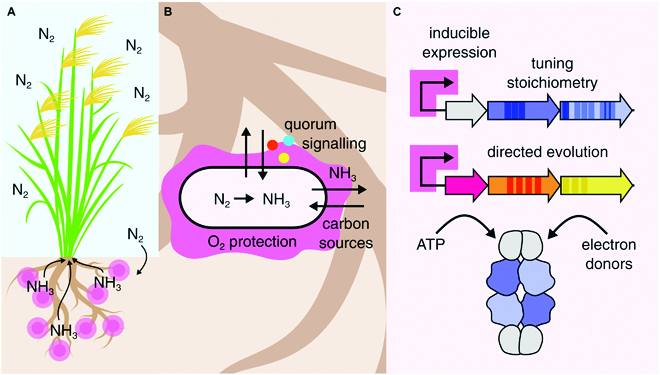
A hypothetical engineered nitrogen-fixing organism promoting wheat growth, highlighting current areas of focus for engineering nitrogenase in synthetic biology. (A) Engineered nitrogen-fixing organisms convert atmospheric nitrogen into ammonia, which promotes plant growth. (B) Engineered organisms exchange fixed nitrogen in the form of ammonia for carbon sources and other nutrients. They may also communicate with the plant through exchange of quorum signaling molecules (colored circles). Artificial oxygen protection systems may be employed to limit oxygen damage to nitrogenase. (C) Approaches used to optimize nitrogenase activity in heterologous contexts include inducible expression or overexpression of nitrogenase genes, tuning stoichiometries of nitrogenase proteins by altering promoters, engineering ribosome binding sites and codon usage, managing supply of ATP and electrons through incorporating additional electron donors, or supplying the organism with high concentrations of sugar, which mimics the exchange of nutrients present in a symbiotic association with plants.

A naive synthetic biology approach for increasing nitrogenase activity in heterologous contexts might be to overexpress nitrogenase genes using constitutive synthetic promoters (Fig. 2C). Diazotrophic organisms express extremely high amounts of nitrogenase under nitrogen-fixing conditions, up to 10% of dry mass in *A. vinelandii*; therefore, one might propose overexpressing all *nif* genes to increase activity [32]. However, it has been repeatedly shown that overexpression of all *nif* genes through the use of strong promoters paradoxically often reduces nitrogenase activity [20,31,33,34]. When *nif* genes (*nifHDK, nifEN, nifJ, nifF, nifBQ*, and *nifUSVWZM*) were expressed under an inducible P_tac_ promoter in *K. oxytoca* knocked out for those genes, some operons (*nifHDK, nifEN*, *nifJ*, and *nifBQ*) showed a clear optimum expression level, with greater expression leading to a rapid loss of nitrogenase activity [33]. Additionally, a reconstructed *nif* gene cluster assembled from *K. oxytoca nif* genes in *E. coli* using an inducible T7 RNAP-based expression system showed the same behavior, with a normal distribution of nitrogenase activity centered on an optimum expression level [35]. While the promoters and organisms differed between these studies, similar relationships were observed between promoter strength and nitrogenase activity. For the catalytic nitrogenase genes (*nifHDK*), nitrogenase activity was generally highest when they were expressed under the strongest promoters; however, this was not true of other nitrogenase genes. Notably, optimal expression levels of *nifUSVWZM, nifEN*, and *nifBQ* fell below the maximum tested, indicating that overexpression of these genes may be associated with cellular toxicity or other effects detrimental to nitrogen fixation. Similar effects were also seen for the complete *nif* gene operon (*nifBHDKENXhesAnifV*) from *P. polymyxa*, where expression under an inducible T7 promoter resulted in a dramatic loss of nitrogenase activity compared to expression under the native promoter [20,34]. Constitutive expression of nitrogenase is also associated with a heavy fitness penalty, creating issues for maintenance of such constructs in chassis species prior to inoculation, since selection pressures will favor deletion of the *nif* genes. These effects may be caused by release of reactive oxygen species by *nif* proteins or depletion of Fe required for other cellular metabolic functions. Nitrogenase overexpression may also result in misfolded proteins, which activate the *E. coli* stress response [36]. Polypeptide stability and solubility is often a barrier for expression of functional nitrogenase in heterologous hosts. Nitrogenase folding is extremely temperature sensitive, with growth at 30 °C showing consistently higher nitrogenase activity than growth at 37 °C, which corresponds to a decrease in the detected level of soluble NifH [35]. Polypeptide stability could theoretically be improved by selecting *nif* genes from thermophilic microorganisms or adaptive laboratory evolution at elevated temperatures (see the “Directed evolution of nitrogenase gene clusters” section). Overexpression may overcome bottlenecks caused by insufficient nitrogenase protein; however, these studies demonstrate that overexpression alone cannot restore nitrogenase activity to similar levels to the original host species. Fine-tuning expression of nitrogenase genes and their regulation is therefore a natural next step to improve nitrogenase activity.

### Tuning individual gene stoichiometries

*Nif* genes are arranged in polycistronic operons that coordinate expression of genes with related functions. *Nif* clusters that are transferred to heterologous contexts lose some of this translational coupling and therefore have altered ratios of *nif* gene products. Changes in stoichiometry between nitrogenase proteins have been proposed to contribute to reduced nitrogenase gene activity when nitrogenase gene clusters are transferred between species [20,31]. Comparing the transcription of *K. oxytoca* transferred into different hosts, the highest nitrogenase activity levels were found in *E. coli*, whose expression of core nitrogenase catalytic genes (NifH, NifD, and NifK) closely correlated with those of the native host [31]. In contrast, those with lower activities differed in their stoichiometric ratios of catalytic Nif proteins [31]. Consequently, it appears that nitrogenase gene activity is highly sensitive to *nif* gene expression ratios, agreeing with the results of previous studies.

Therefore, we may expect that controlling expression of each individual *nif* gene to optimize their stoichiometries is a straightforward engineering target that should result in an increase of nitrogenase activity (Fig. 2C). This has motivated the design of several studies that seek to rationally tune expression levels of core nitrogenase genes, to restore optimum nitrogenase enzyme stoichiometries (which resemble those in the native nitrogen-fixing species). Despite intense efforts in synthetic biology to achieve predictable control of gene expression through the careful choice and rational optimization of promoter and ribosome binding site (RBS) strength, gene expression is highly sensitive to genetic and metabolic host contexts; therefore, achieving fixed ratios of gene expression in genetic circuits is a complex task [37]. In silico tools such as the RBS calculator and RBSDesigner, which rely on a thermodynamic model and comparison to an optimal Shine–Dalgarno sequence to predict expression of genes in different hosts, have allowed rational tuning of RBS strengths in designed metabolic pathways [38,39]. This approach has succeeded in other metabolic engineering projects, especially combined with a straightforward screening and selection protocol for improved variants [40]. Nonetheless, a further obstacle is that quantifiable rules for linkages between expression ratios of *nif* genes and activity remain elusive.

In one exhaustive study, *nif* genes from *K. oxytoca* were refactored by codon optimizing and placing them under control of synthetic regulation; libraries were constructed where both the order and direction of transcriptional units were varied using a combinatorial assembly approach, to identify clusters with increased activity [41]. Recovered operon architectures with increased activity showed variability in gene order and expression levels, with considerable variability in growth rates. Importantly, activity was increased further, to 57% of wild-type activity in *K. oxytoca*, by introducing and screening RBS libraries upstream of *nifH*, *nifD*, and the *nifENJ* operon and including *nifUSVWZM* genes in the cassette. Expression analysis of this cluster revealed that while transcript levels varied greatly from those of the native cluster, absolute expression (as measured by proteomics) had almost identical gene expression ratios to the original heterologous *K. oxytoca* gene clusters [41]. Therefore, one important conclusion from that study is that absolute expression levels of clusters are generally well-optimized in the native host, and therefore, further tuning of RBS sequences is unlikely to improve nitrogenase activity, except where there are significant differences in the mRNA binding region of the 16S rRNA, or the cellular environment between the original and host species.

When these reshuffled clusters were transformed into *E. coli*, the activity of the cluster that had the highest activity in *K. oxytoca* dropped to only 7% of the activity of the original cluster in *K. oxytoca*. Indeed, the best-performing construct in *E. coli* retained only 18.5% of the activity of the wild-type cluster in *K. oxytoca* [41]. Later development of this cluster replaced the operons in the refactored cluster with the native operons, while retaining the refactored design of the other genes. This reduced activity in *E. coli* permitted nitrogen fixation activity in *Rhodobacter sphaeroides* IRBG74 and *Pseudomonas protegens* Pf-5. The latter activity was not present when the original cluster was transferred [31]. From these successive iterations and transfers, we can see that optimizations of stoichiometries in one host often do not easily translate to improvements in absolute measured activity in another, despite very similar host physiologies. Furthermore, there are many potential solutions in sequence space that produce equal nitrogenase activity, despite very different operon architectures and expression levels.

An alternative approach that is less sensitive to differences in RBS and promoter strength differences between hosts is using translational fusions. A recent study aimed to simplify regulation of the *K. oxytoca* nitrogenase cluster by creating 5 “giant” gene fusions from 14 individual *nif* genes, either translationally fused with flexible linkers or post-translationally cleaved. Fixing expression ratios between these genes in this way showed remarkable success in improving nitrogenase activity, with engineered *E. coli* strains exhibiting 51% of the original wild-type nitrogenase activity, and supporting growth in nitrogen-limited minimal medium [42].

This work highlights that merely tuning RBS and promoter strengths to match those of the native host does not result in predictable increases in nitrogenase cluster activity. The lack of predictability of nitrogenase activity from expression levels alone is compounded by the high degree of translational coupling between *nif* genes and feedback inhibition through cellular nitrogen status.

### Inducible expression in response to fixed nitrogen and oxygen

In nature, nitrogenase protein expression is tightly regulated in response to nitrogen availability and oxygen concentration, avoiding expression of metabolically expensive nitrogenase genes in unsuitable conditions [13]. Heterologous *nif* clusters display one of two potential regulatory phenotypes. Either they are constitutively active, and insensitive to the presence of fixed nitrogen, owing to incompatibility of absence of regulatory proteins, or they respond similarly to the native organism [5,20,22,30,31,34]. Despite control of expression being a crucial component of the lifestyles of diazotrophic organisms, heterologous *nif* genes have rarely been expressed under nitrogen and oxygen responsive promoters, at least past the initial stages of testing of nitrogenase gene clusters. The reason for this is to avoid complicating the design process with additional regulatory feedbacks. In some cases, regulation in response to fixed nitrogen is considered undesirable, and organisms have been genetically engineered to remove this limitation. For example, recently, the first commercially available microbe for corn has been developed, based on *Klebsiella variicola* strain 137 that expresses *nif* genes constitutively, resulting in a dramatic increase in nitrogenase activity under nitrogen-rich (fertilized field) conditions [43]. While clusters with the native promoters often show higher nitrogenase activity in heterologous hosts compared to stronger constitutive or inducible systems under nitrogen-fixing conditions, this aspect of operon engineering has not yet been fully explored for its potential to reduce the metabolic burden of nonfunctional nitrogenase expression under real-world conditions [5,20,30,31,34]. In a hypothetical engineered nitrogen-fixing organism, survival in an agricultural environment should depend on responding to external signaling cues to regulate nitrogenase expression. This is important because nitrogen fixation is extremely energetically costly, and therefore, cells expressing nitrogenase will encounter a fitness penalty in a competitive soil environment. Consequently, inducible expression under relevant conditions is critical to ensure survival of engineered nitrogen-fixing organisms.

### Engineering symbiotic relationships

Many nitrogen fixers participate in mutualistic associations with legume crops, forming rhizobial biofilms or root nodules that facilitate the exchange of carbon, protect the nitrogen-fixing bacteria from oxygen, and exclude competing species [13,44,45]. Engineering symbiotic relationships between synthetic soil bacteria and cereal crops involves establishing mutual dependencies, either through engineering plants to express orthogonal signaling molecules, which activate *nif* gene expression or ammonia secretion, or alternatively by engineering species to respond to existing chemical signals present in the crop plant rhizosphere [46]. Mutualistic associations are established through exchange of plant-produced sugars, fixed nitrogen, and diffusible quorum sensing molecules between the plant and the microorganism [45]. The most complex of these microenvironments, root nodules, employ oxygen gradients, leghemoglobin, local oxidases, and physical separation to create an anaerobic or microaerobic environment where nitrogen fixation can occur [13,44,45]. Engineering nodulation in cereal crops is a huge technological challenge that is currently beyond the field’s scientific capabilities. Looser associations may involve free-living soil bacteria contributing fixed nitrogen to the surrounding soil, but remaining distinct from the plants themselves, or by colonizing mucilaginous or alginate biofilms covering the plant roots [47–49]. Even simpler symbiotic relationships are possible: *nif* promoters could be engineered to respond to orthogonal chemical signals secreted by plants. This would ensure *nif* genes are only expressed close to roots, while reducing the fitness burden of expressing nitrogenase under nonsymbiotic conditions. Alternatively, orthogonal quorum sensing molecules could be used for which bacteria already have well-characterized sensors; however, this has the limitation of requiring the plant to be engineered to produce this signal [50]. Rhizopene has been identified as a potential signaling molecule, whose synthesis pathway can be engineered into transgenic crops, excreted into the root rhizosphere, and be detected by genetically encoded biosensors in rhizobia [51]. A rhizopene-producing transgenic barley line was used to establish synthetic plant–microbe signaling circuit between barley and *Azorhizobium caulinodans* ORS571, a model cereal endophyte. In this work, the gene encoding NifA, the master regulator of nitrogenase genes in many species (see the “Genetic regulation of *nif* clusters” section) was placed under the control of a rhizopene-inducible promoter. This permitted limited exogenous control over *nif* gene expression and nitrogen fixation activity; however, many bacteria did not respond to the chemical signal; therefore, more work is needed to develop rhizopene biosensors that are stable in the soil environment [52]. Natural symbiosis relies on nutrient exchange in addition to small-molecule signaling, which mitigates the high metabolic costs of nitrogen fixation. In this rhizopene-based system, the bacterium receives no benefit from expression of energetically costly nitrogenase genes; hence, there is a strong selection pressure to “escape” by gene silencing or mutation. Combining an orthogonal signaling system with nutrient exchange between engineered bacteria and transgenic plants will therefore be crucial to support bacterial growth and therefore establish a stable plant–bacteria engineered symbiotic relationship. When working with synthetic *nif* operons in heterologous hosts, nitrogenase gene expression must be tuned to accommodate the lifestyle of the heterologous organism to minimize metabolic cost and support the maintenance of the diazotrophic species in the rhizosphere. Using the strategies described above, we may mitigate competition from organisms with faster growth rates in the soil environment and thus engineer true symbiotic relationships between engineered nitrogen-fixing organisms and cereal crops.

### Engineering with electron carriers

Nitrogenase requires a source of electrons to provide reducing power for atmospheric nitrogen reduction. Fortunately, NifH is remarkably promiscuous, accepting electron transfer from bacterial, cyanobacterial, and mitochondrial reduced electron donors (ferredoxin and flavodoxins) [53]. Many gene clusters are associated with electron transport to nitrogenase in different species (see Table 1). The presence of nitrogenase activity in *E. coli* transformed with a minimal *nif* gene cluster, which does not include electron carriers, indicates that nitrogenase can accept electrons from electron carriers encoded on the *E. coli* genome [20]. However, managing electron supply to heterologous *nif* operons has the potential to increase activity by increasing the supply of high-energy sources of electrons for the heterologous nitrogenase proteins such as those from aerobic respiration [5,22,54]. Electron sources could be oxidation of H_2_ or pyruvate, central metabolism, or cyclic electron pathways provided they have sufficient reducing potential [22].

**Table.** Synthetic biology “toolbox” of core and additional genes associated with nitrogenase activity that could be used in synthetic biology contexts. Synthetic biology projects involving nitrogenase gene clusters may select parts from this toolbox to improve electron transport, metallocluster biosynthesis and maturation, and oxygen protection and regulation in heterologous host contexts.

**Function**	**Gene names**	**Examples of nitrogen-fixing species containing gene**	**Reference**
Nitrogen fixation (MoFe)	*nifH, nifD, nifK*	N_2_ fixing species	
Nitrogenase cofactor biosynthesis	*nifE, nifN*	N_2_ fixing species	
Nitrogenase cofactor biosynthesis	*nifB, nifX*	N_2_ fixing species	
Nitrogen fixation (FeFe)	*anfH, anfD, anfK, anfG*	*Azotobacter vinelandii*	[80]
Nitrogen fixation (VFe)	*vnfD, vnfK, vnfG*	*Azotobacter vinelandii*	[80]
Homocitrate synthase	*nifV, lys20*	*Azotobacter vinelandii, Klebsiella oxytoca M5a1, Thermus thermophilius HB27*	[21,80]
Nitrogenase cofactor biosynthesis	*iscA, lrv, nifZ, nifQ, nifW, nifY, nifO, nafY*	*Azotobacter vinelandii, Rhodobacter capsulatus, Klebsiella oxytoca*	[80,81]
Fe-S cluster biosynthesis—SUF	*nifS, nifU, sufA, sufB, sufC, sufD*	*Klebsiella oxytoca, Azotobacter vinelandii*	[80]
Fe-S cluster biosynthesis—ISC	*iscA, iscB, iscS, iscR*	*Paenibacillus polymyxa*	[54,82]
Posttranslational processing	*nifM, clpX*	*Azotobacter vinelandii*	[53,83]
Oxidoreductase	*nifJ*	*Azotobacter vinelandii, Klebsiella oxytoca M5a1*	[84]
Electron transport	*rnf* genes	*Rhodobacter capsulatus*	[85]
Electron transport	*fix* genes	*Klebsiella oxytoca, Rhodobacter Rubrum, Azotobacter vinelandii*	[86]
Electron transport	PFOR genes	-	[22]
Electron transport	*fer* genes, *nfrA*, COG3411	*Paenibacillus polymyxa*	[22]
Ferredoxin	*fdxA, fdxB*	*Azotoacter vinelandii*	[22]
Ferredoxin	*fdxE, fdxC*	*Rhodobacter capsulatus*	[22]
Flavodoxin	*nifF, fldA, fldB, cpFld1, cpFld2, cpFld3*	*Escherichia coli, Rhodobacter capsulatus, Azotobacter vinelandii, Klebsiella pneumoniae, Clostridium pasteurianum*	[22]
Oxygen protection	*FeSII* (Shethna protein)	*Azotobacter vinelandii*	[87]
Oxygen protection	*nafU*	*Azotobacter vinelandii*	[28,66]
Unknown	*nifT*	*Klebsiella oxytoca, Azotobacter vinelandii*	[80,81]
Unknown	*hesA*	*Paenibacillus polymyxa*	[20,88]
Unknown	*orf1*	*Paenibacillus graminis*	[89]

Deletion analysis of constructs containing iron-only nitrogenase structural genes, accompanied by *K. oxytoca* biosynthetic and electron transport genes in *E. coli* showed that the Fe-only nitrogenase has different gene requirements to the MoFe nitrogenase in *E. coli* [5]*.* Essential genes included *nifF* (encoding an electron carrier) and *nifJ* (encoding an oxidoreductase) in *K. oxytoca*, whose functions can be partially substituted by native *E. coli* electron carriers but cause a dramatic reduction of nitrogen fixation activity when deleted from the recombinant strain [5]. This finding was confirmed in a follow-up study from the original *Paenbacillus WLY78* transfer to *E. coli*, where coexpression of *nifF* and *nifJ* genes from *K. oxytoca* increased nitrogenase activity from 10% of wild-type activity to 32% [54]. Coexpression of potential electron transporters (*pfoABfldA*) together with the *nifSU* operon from *K. oxytoca* from *Paenibacillus* further increased activity to 50% [54]. Further examples of gene clusters related to electron transfer in nitrogenase can be found in Table 1. These results demonstrate that inefficient electron transfer to nitrogenase may limit nitrogen fixation activity in heterologous hosts. In addition to electrons, nitrogenase activity is dependent on abundant intracellular ATP supply, which is the next engineering consideration.

### Engineering ATP supply

Maintaining high ATP supply to nitrogenase is required for high nitrogen-fixation activities. Nitrogen fixation has a high ATP requirement, but requires anaerobic or microaerobic conditions. This presents a paradox, as metabolism under anaerobic conditions (anaerobic respiration or fermentation) produces less ATP than aerobic respiration [55]. In nature, ATP required for nitrogen fixation is provided through a variety of mechanisms (see Introduction). Typical strategies employed to meet the energy requirement of nitrogenase are either supplying high sugar content in the growth media (the most common approach in synthetic biology studies) or supplying energy through an aerobically respiring partner (either a plant or another microorganism). In a hypothetical plant–microbe engineered symbiotic relationship, exchange of sugars would ideally occur alongside exchange of diffusible signaling molecules, allowing nitrogen-fixing cells to maintain the high metabolic rate required for nitrogenase activity.

### Engineering oxygen protection

Proposed mechanisms of protecting oxygen in a synthetic nitrogen-fixing plant often mimic oxygen protection strategies found in nature. These mechanisms can be divided into four broad categories, which will be described below.

### Physical protection

Many species use physical protection strategies to maintain nitrogenase activity and avoid oxygen damage. Root nodules are colonized by rhizobia bacteria, which depend on the legume for nutrients while providing the legume a local source of fixed nitrogen [56]. Legumes avoid oxygen damage to nitrogenase by expressing leghemoglobin, which binds oxygen to create a local microaerobic environment. Copying this strategy, bacterial hemoglobins or FeSII protein (see Table) could be engineered into plant roots or expressed in an engineered microbe, acting as an oxygen sink surrounding domains of nitrogenase activity to protect enzyme activity in low (but not zero)-oxygen conditions.

Biofilms are important for facilitating symbiotic relationships and maintaining microaerobic or anaerobic conditions for diazotrophic bacteria. Many nitrogen-fixing organisms, including *Azotobacter* and *Klebsiella*, secrete large amounts of mucus or alginate that form a physical barrier to oxygen penetration [44,48,57]. Thus, genes for mucus biosynthesis and secretion may be straightforward to incorporate in engineered strains of bacteria or genetically engineered crops. Similarly to nitrogenase gene clusters, the majority of genes for alginate biosynthesis in *A. vinelandii* are found in a single operon, which may facilitate pathway import into engineered diazotrophic bacteria [58].

Mimicry of these strategies presents intriguing possibilities for synthetic biology projects. For instance, coexpression of biofilm-producing enzymes could protect nitrogenase from oxygen using the mechanism of oxygen protection employed by many free-living and root-associated bacteria. It is worth noting that work so far to increase oxygen tolerance of nitrogenase has mainly focused on measurements performed in liquid culture, where the effects of biofilms are reduced; thus, this presents an unexplored opportunity.

### Conformational protection

Conformational protection occurs at the enzyme level in *A. vinelandii* through reversible binding of FeSII, also known as Shethna protein, an oxygen protective factor that associates with NifHDK, forming an oxygen-resistant but catalytically inactive state [59]. In this way, FeSII could be employed to conformationally protect nitrogenase in an engineered strain, reducing nitrogenase damage when molecular oxygen rises, and therefore reducing the metabolic cost of replacing oxygen-damaged nitrogenase.

### Temporal regulation

In some species of bacteria, temporal regulation is used to reconcile the paradox of photosynthesis (which is essential for life) producing oxygen, while oxygen damages nitrogenase (which is also essential). During the night, when photosynthetic oxygen production does not occur, nitrogenase is expressed, using stored glycogen as an ATP source [60,61]. Hence, cyclic expression of nitrogenase genes corresponds to the nitrogen fixing activity of the cyanobacteria at different times of day [62]. In the laboratory, one could envisage using destabilized nitrogenase constructs with short half-lives (LVA tags) that are expressed either continuously or cyclically to replenish active nitrogenase at times of suitable low oxygen exposure [63].

### Respiratory protection

Increasing respiratory rate can also protect oxygen-sensitive nitrogenase subunits, by locally lowering oxygen levels. *A. vinelandii* achieves this primarily through the expression of 5 terminal oxidases and several parallel respiratory pathways [28]. Furthermore, cyanobacterial heterocysts protect nitrogenase by increasing their respiratory rate, which lowers local oxygen concentration to microaerophilic levels [13]. Again, such strategies might be exploited in laboratory projects, to engineer synthetic constructs in modified bacteria to protect engineered nitrogenase genes from molecular oxygen.

### Alternative oxygen-protection strategies

In addition to the categories described above, other approaches may be used to insulate NifHDK from molecular oxygen. For example, coexpression of uptake hydrogenase reduces nitrogenase oxygen sensitivity in many species [64]. In one study, an uptake hydrogenase from diazotrophic cyanobacterial species *Cyanothece* 51142 was coexpressed with nitrogenase genes in a nondiazotrophic cyanobacterial species *Synechocystis* sp. PCC 6803 to enhance oxygen tolerance, thereby improving nitrogenase activity [65]. A novel oxygen-responsive gene, *nafU*, was recently identified in a transcriptomic analysis of *A. vinelandii* whose expression depended on oxygen concentration. Overexpression of *nafU*, encoding an inner membrane protein, in *A. vinelandii* increased nitrogen fixation activity under aerobic conditions (20% oxygen). Furthermore, *nafU* expression in *E. coli* increased heterologous nitrogenase activity under aerobic conditions [66]. These strategies may be generally applicable to engineering nitrogenases in heterologous organisms. In addition to protecting nitrogenase from oxygen, understanding the soil microenvironment surrounding crop plants is essential to mitigate competition between engineered nitrogen-fixing bacteria and other microorganisms present in the soil, which are likely to be slow-growing, due to the high energy demands of nitrogenase synthesis and nitrogen fixation. The ecological requirements for analogous engineered species are all likely to be complex, which we will discuss further in the following section.

### Directed evolution of nitrogenase gene clusters

Finally, we will explore how directed evolution may be used to select nitrogenase variants with improved activity. Directed evolution has been successful in evolving small-molecule sensors, optimized promoters, and enzymes with novel substrate recognition capabilities for both biotechnological and synthetic biology applications [67–71]. Initial sequences may be diversified in vitro through error-prone PCR, combinatorial assembly, or site-directed mutagenesis, or in vivo through use of mutant or native error repair or DNA replication methods, or even natural background genomic mutation to generate sequence diversity in living cells [72–77]. In nitrogenase, which includes many operons and different gene clusters, library generation is time-consuming and costly, as the combinatorial space of library variants is massive. Sequence variants in directed evolution experiments may be assayed through either screening or selection. In a typical screening approach, desired protein activity is coupled to a measurable signal, such as expression of a fluorescent protein, which may be amenable to high-throughput analysis. In a selection-based approach, cells are grown under conditions that give a selection advantage to cells carrying genes with more desirable properties. Applying a selection pressure restricts survival of cells containing inactive nitrogenase variants and permits growth of more active variants, enriching them within a mixed culture. In nitrogenase selection systems, more active nitrogen-fixing variants should show a clear growth advantage when cells are grown under nitrogen-free or low-nitrogen conditions. In general, considerable efforts have been directed toward minimizing “cheaters” in mixed-population selection experiments, where cells may “escape” the selective additions by generating unrelated mutations that permit survival. Nitrogenase has an advantage in this regard, since there is only a small margin for adaptation to low fixed-nitrogen conditions. However, *E. coli* cells themselves contain between ~11% and 13% nitrogen content in dry mass; therefore, the cell density is also an important factor in determining selection pressure in a selective system to prevent survival by nitrogen scavenging rather than nitrogen-fixation-dependent growth [78].

Building a library of nitrogenase clusters with variable regulatory elements in *E. coli* was previously only possible through a hierarchical combinatorial assembly approach, yielding clusters that varied the order and expression levels of *nif* genes. From this, some useful insights into the preferred ratios of particular genes within this system were obtained, despite recovered operons being highly structurally diverse [41]. While the assembly method for this was high-throughput, screening of the variants was performed using a low- to medium-throughput standard acetylene reduction assay. Since a medium-size RBS library has not identified any variants with close to the original nitrogenase activity of engineered clusters, this indicates that the sequence space of nitrogenase gene clusters is sparsely occupied. Successful evolution of nitrogenase will therefore likely rely on accessing a broader sequence space and using a strong selection pressure that is able to discriminate clearly between populations with different activities.

Alternatively, libraries may focus on mutating the coding sequence, with the aim to increase activity or increase compatibility with other host enzymes, or to select a desired enzymatic activity. In one study, Barahona and colleagues screened a library of NifH variants for hydrogen production in a strain of *Rhodobacter capsulatus* that was “knocked out” for uptake hydrogenase. Nitrogen fixation produces H_2_ as a (normally undesirable) by-product, and therefore, diazotrophic species employ an uptake hydrogenase to convert the excess hydrogen into protons and recover electrons. However, hydrogen production may be desirable for applications such as generating renewable hydrogen biofuels. Building a fluorescent reporter for H_2_-responsive expression permitted screening of more active variants by fluorescence-activated cell sorting, allowing quick identification of nitrogenase variants producing more H_2_ and less ethylene in acetylene reduction assays. The identification of a high-throughput assay for hydrogen production is a powerful approach for screening large libraries of nitrogenase variants. This remains one of the only studies to date that modifies nitrogenase on the sequence level to modulate activity [79].

Screening and selection approaches are further complicated by the fact that many nitrogenase gene clusters are regulated by cellular status feedback as discussed previously and the contribution of cellular biomass to fixed nitrogen availability in a nitrogen-limited medium. Alongside modification of the clusters themselves, mutation, activation, or repression of endogenous genes relevant to nitrogenase could also be used alongside to improve compatibility of heterologous *nif* operons with their hosts. Finally, to select improved nitrogenase operons, selection pressures should be quantifiable within a given system, which will allow close control of selection pressure during an evolution experiment.

### Future outlooks

Through the work outlined in this review, synthetic biologists are developing an understanding of different factors that contribute to nitrogenase activity and compatibility with hosts. A few important discoveries have been made through designing these systems from the bottom-up, starting by looking at nitrogenase gene clusters in different branches of evolution.

Nitrogenase is found in many diverse prokaryotes, as a result of horizontal gene transfer, and is often organized into tightly associated operons and gene clusters. This indicates that nitrogen fixation may be compatible with many host physiologies, making it a promising target for integrating into new hosts. Furthermore, enzymes from remarkably different species can function together to fix nitrogen, making the minimal gene set shown above modular and flexible. Operon architecture, while highly coupled between certain genes, may vary widely while retaining nitrogenase activity. An engineering approach to implementing this coupling is to use either gene fusions or feedback control of RBSs. This has the additional advantage of reducing the number of variants to screen for optimum gene stoichiometries and operon architectures, allowing the discovery of more active variants more easily in different hosts.

While minimal gene clusters are often sufficient to achieve nitrogen fixation activity, compatibility between nitrogenase genes and heterologous hosts may be increased by either modifying functional orthologs already present in the host species or incorporating additional genes that increase compatibility, thereby increasing activity. This is particularly important in the case of electron transfer to NifH, which may vary significantly due to different electrophysiological environments in different hosts. Nitrogenase requires specific metallocluster cofactors that have complex synthesis pathways. These synthesis pathways differ in different contexts, and reengineering of host metabolic pathways may be necessary to deliver the required substrates.

Energy supply to fulfill the ATP requirement of nitrogenase remains an important obstacle to be overcome. Most studies to date have measured nitrogenase activity in heterologous hosts, in high-nitrogen and high-glucose media, with very few discussing how these conditions might be achieved in the context of an agricultural soil environment. Presumably, engineering a symbiosis between plants and bacteria may somehow include secretion of sugars to fuel the energy requirement of nitrogenase, although tuning this is not straightforward to say the least.

Survival of engineered bacteria in real-world environments, including engineering symbiosis, has not yet been explored in detail. While *E. coli* is a good chassis species for exploring the factors relating to successful optimization of heterologous nitrogenase clusters, much work needs to be done toward engineering synthetic symbioses that survive competition with other soil microorganisms. Alternatively, engineered bacterial endophytes that colonize plant tissue directly could be created. This is an even more challenging engineering problem, as hundreds of genes are likely to be involved in the colonization of plants.

Finally, directed evolution might also be applied to nitrogenase operons in heterologous hosts. This is a challenging approach for several reasons. Firstly, achieving high library diversity of engineered nitrogenase variants to screen is difficult because the application of library generation methods for *nif* operons is limited by their complexity and size. Moreover, expression of nitrogenase genes induces high metabolic burden and leads to growth defects in host cells. Particularly challenging is the fact that the sequence and phenotypic space of nitrogenase genes is extremely large, meaning that achieving sufficient representation of library diversity requires large cultures. Simplified or minimal nitrogen fixation gene clusters may be used to generate more focused libraries as described above, reducing experimental complexity. Additionally, screening this sequence space for nitrogenase is time-consuming and low-throughput using the standard acetylene reduction assay used to quantify nitrogen-fixation activity. Fortunately, low fixed-nitrogen availability exerts a strong selection pressure for cell survival under low fixed-nitrogen conditions, and therefore, we might foresee that, if carefully controlled, this could be exploited to create an evolutionary trajectory that selects for more active nitrogenase variants.

Whichever approach is used, improving nitrogenase activity toward nitrogen-fixing plants is a complex and challenging engineering problem, which will require creative solutions as varied and dynamic as those we see in the natural world.
